# Obstructive Sleep Apnea in Neurodegenerative Disorders: Current Evidence in Support of Benefit from Sleep Apnea Treatment

**DOI:** 10.3390/jcm9020297

**Published:** 2020-01-21

**Authors:** Annie C. Lajoie, Anne-Louise Lafontaine, R. John Kimoff, Marta Kaminska

**Affiliations:** 1 Respiratory Epidemiology and Clinical Research Unit, Research Institute of the McGill University Health Centre, Montreal, QC H4A 3S5, Canada; annie.lajoie@mail.mcgill.ca (A.C.L.); r.kimoff@mcgill.ca (R.J.K.); 2 Montreal Neurological Institute, McGill University Health Centre, Montreal, QC H3A 2B4, Canada; anne-louise.lafontaine@mcgill.ca; 3 Respiratory Division & Sleep Laboratory, McGill University Health Centre, Montreal, QC H4A 3J1, Canada

**Keywords:** obstructive sleep apnea, neurodegenerative diseases, Parkinson’s disease, Alzheimer’s disease

## Abstract

Obstructive sleep apnea (OSA) is a prevalent disorder characterized by recurrent upper airway obstruction during sleep resulting in intermittent hypoxemia and sleep fragmentation. Research has recently increasingly focused on the impact of OSA on the brain’s structure and function, in particular as this relates to neurodegenerative diseases. This article reviews the links between OSA and neurodegenerative disease, focusing on Parkinson’s disease, including proposed pathogenic mechanisms and current knowledge on the effects of treatment.

## 1. Introduction

Obstructive sleep apnea (OSA) is a treatable sleep respiratory disorder characterized by recurrent partial (hypopnea) or complete (apnea) upper airway obstruction during sleep, resulting in intermittent hypoxia and sleep fragmentation. It is a common disease, especially in the aging population, with a prevalence of nearly 60% and 40% of men and women over 60 years old, respectively [1]. OSA is associated with excessive daytime sleepiness and various neurocognitive and psychological manifestations, such as depression [2]. Over the past few years, there has been growing interest in the impact of OSA on brain structure and function, especially in the elderly population and in patients with neurodegenerative diseases. One systematic review reported that patients over 40 years old suffering from OSA were 26% more likely to develop signs of cognitive decline or dementia [3]. Furthermore, another cohort study found that individuals with sleep disordered breathing developed mild cognitive impairment or Alzheimer’s disease (AD) related dementia at a younger age [4].

Neurodegenerative diseases such as AD and Parkinson’s disease (PD) are increasingly prevalent with advancing age. PD, which is characterized by its hallmark motor features of tremor, bradykinesia, rigidity, and postural instability, together with important non-motor symptoms including cognitive dysfunction, is the second most frequent and the fastest growing neurodegenerative disease [5,6]. Sleep disturbances are frequently found in PD, where the prevalence is estimated to be as high as 60% to 90% [7,8]. These include alterations of sleep architecture, insomnia, hypersomnia, restless legs syndrome, rapid-eye movement sleep behaviour disorder (RBD), which can precede the appearance of motor symptoms, and sleep-related breathing disorders [9,10,11,12]. It is estimated that 20% to 60% of PD patients have concomitant OSA [13]. Whether PD increases OSA prevalence is still largely debated. Nevertheless, there is biologic plausibility for PD to be involved in the pathogenesis of OSA. Conversely, OSA appears to have a detrimental impact on brain structure and function. Thus, when already affected by a neurodegenerative process, the brain could be more vulnerable to the additional effects of OSA. 

In this article, we review the interactions between OSA and neurodegenerative diseases, with a particular focus on PD. We explore proposed pathophysiological mechanisms and the possible impact of treatment on the clinical evolution.

## 2. The Consequences of OSA on the Vulnerable Brain

### 2.1. OSA and Cognitive Function in the General Population

In the general population and the elderly, OSA appears to be associated with impaired cognition and psychomotor performance [14]. Numerous studies have specifically sought to assess the effects of OSA on global cognition and specific neurocognitive domains. Systematic reviews and meta-analyses have reported relatively consistent deficits in attention, vigilance and executive functions, occasional impairment of some subdomains of memory, and relative sparing of visuospatial abilities, language abilities, psychomotor function, short-term memory, and global cognition [3,15,16]. However, the evidence is sometimes contradictory since OSA has not been universally found to lead to cognitive dysfunction and no clear relation has been found between OSA severity and the propensity for cognitive impairment. Indeed, one systematic review found that, apart from attention and vigilance, more severe OSA did not produce a greater impact on other cognitive domains as compared with milder OSA [17]. These discrepancies could, in part, be due to methodological factors such as often low sample sizes and incomplete accounting by studies and reviews of potential confounders, such as level of education or ethnicity, and variable outcome measures [15]. Studies use a wide and inconsistent array of tests to assess each cognitive domain and there is considerable overlap between domains within each test. Another possible explanation is one of cognitive reserve where individuals with greater premorbid cognitive functioning are less vulnerable to sustained or repetitive brain injury, and thus less likely to develop cognitive dysfunction than their cognitively vulnerable counterparts given the same insults [18]. The effect of sleep disturbances appears to be modified by cognitive reserve for at least some cognitive domains [19]. Furthermore, due to the chronicity of OSA, adaptive cerebrovascular mechanisms resulting from repetitive exposure to intermittent hypoxemia, such as development of vascular collaterals or changes in the regulation of cerebral blood flow, could lessen the adverse impacts of OSA and further mitigate the development of cognitive dysfunction [20]. Moreover, effects of OSA on the brain can differ depending on age, gender, comorbidities, and variable definition of OSA [21,22]. 

### 2.2. OSA and Dementia

The aging brain is more prone to cognitive impairment. Alzheimer’s disease (AD) is the most common form of dementia and its prevalence increases with age. Prospective studies have demonstrated that older individuals with OSA at baseline were more likely to develop cognitive impairment and, subsequently, evolve towards frank dementia at follow up [3,4,23,24,25]. Amongst elderly women, the presence of OSA was associated with increased odds of developing subsequent mild cognitive impairment (MCI) and dementia (OR: 1.85; 95% CI: 1.11 to 3.08) after adjustment for potential confounders [23]. This is supported by a nationwide database study from Taiwan that found a two-fold increase in risk of dementia in patients with OSA and that female, unlike male subjects, were more likely than non-OSA controls to suffer from dementia [24]. A study of the Alzheimer’s Disease Neuroimaging Initiative cohort found that patients with sleep disordered breathing developed MCI or AD-related dementia about 10 years earlier than patients without OSA [4]. More recently, the ARIC (Atherosclerosis Risk in Communities) cohort study also found that severe OSA was associated with an increased risk of all-cause and AD-related dementia [26]. However, the association was reduced when adjusted for cardiovascular risk factors. This is of importance, as neurodegeneration could also result from cerebrovascular effects of OSA. Indeed, OSA can induce sympathetic activation and endothelial dysfunction and predispose to hypertension and cerebrovascular diseases, such as vascular dementia [27]. Looking at individuals with an AD diagnosis, a meta-analysis of cross-sectional studies found that AD patients had five times the odds of OSA as compared with healthy controls and it was estimated that OSA occurred in nearly 50% of AD patients [28]. Although this does not imply directionality or causality, it highlights the importance of the relationship between OSA and AD. 

Recent epidemiological studies have provided evidence that OSA could act as a precursor to other neurodegenerative diseases, such as PD. Indeed, four retrospective nationwide registry-based cohort studies from Taiwan have evaluated incident PD in patients with OSA [29,30,31,32]. All four studies found an increased incidence of PD as compared with non-apnoeic controls. The sex-related risk varied between studies as two of these studies found the risk to be present in males only [29,30], one in females only [32], and one in both males and females [31]. The results suggest that OSA could be involved in the pathogenesis of neurodegenerative diseases, although it is also possible that OSA is more likely to be diagnosed in preclinical PD given that both are associated with disrupted sleep.

### 2.3. OSA and Cognitive Function in PD

Non-motor symptoms (NMS) are prevalent in PD, even early in the disease course and have a negative impact on health-related quality of life [33]. Among these, cognitive dysfunction is estimated to be present in 205 to 40% of patients with early PD and up to 47% with normal baseline cognition develop cognitive impairment at six years follow up [34,35]. A study conducted by our group found that cognitive dysfunction, measured using the Montreal Cognitive Assessment (MoCA), was greater in PD patients with concomitant OSA as compared with PD patients without OSA and that the impairment increased with OSA severity [36]. Other groups that looked at global cognitive function measures have found similar results [37,38,39]. Specific domain most often affected were attention and vigilance, visuospatial abilities, and executive function [39]. However, other studies did not reach the same conclusions [40,41]. In the first study, 92 PD patients were screened for OSA and submitted to a complete neuropsychological assessment, including MoCA [40]. However, the number of patients with PD and OSA was fairly small and represented only 21% of patients. In another study, 100 PD patients (50 unselected and 50 sleepy patients) were evaluated to assess the frequency of OSA and the impact of RBD on the severity of OSA [41]. There was no significant difference between groups in terms of global cognition, as measured by the mini-mental state examination (MMSE). However, the MoCA is thought to be more sensitive for cognitive dysfunction in PD. Moreover, the prevalence of OSA was relatively low (27% of PD patients versus 40% of controls) in this study. Thus, discrepancy could result from differences in patient populations, for example lower prevalence and severity of OSA or of cognitive dysfunction, different target populations for study recruitment (e.g., focused primarily on RBD), and differences in OSA definitions and scoring practices which can impact OSA detection [42,43]. 

### 2.4. OSA and Motor Impairment in PD 

Motor impairment is a prominent feature of PD and results from degeneration of dopaminergic neurons in the substantia nigra and depletion of dopamine from the basal ganglia, areas of the brain involved in motricity control. This manifests clinically by bradykinesia, rigidity, tremor, and postural instability, as well as gait disturbances. In the general population, OSA has been found to be associated with falls and disturbed gait, which improved with treatment of OSA [44,45,46]. Interestingly, although gait has been considered an automatic motor function, a cognitive component is now believed to be involved, particularly executive function [47]. The interaction of OSA and motor dysfunction in PD has been less extensively studied. Motor dysfunction in PD is often clinically measured with the MDS-UPDRS (Movement Disorder Society Unified Parkinson’s Disease Rating Scale), a four-part questionnaire that assesses experiences of daily living, motor symptoms, non-motor symptoms, and motor complications [48]. A higher score indicates greater impairment. The Timed Up-and-Go (TUG) is a test used to assess basic mobility, balance, and risk of falls in the elderly [49]. A prospective cohort study conducted by our group showed that PD patients with OSA had significantly higher baseline motor MDS-UPDRS [50]. This was consistent with other studies in which motor function was worse in PD patients with OSA [41]. Furthermore, those with OSA treated with CPAP showed a stabilization of their motor MDS-UPDRS scores over a 12-month follow-up period, whereas in PD patients without OSA and those with untreated OSA, motor scores deteriorated. In this study, we used a model where CPAP use (in hours per night of use) was treated as a continuous variable. We found that progression of motor symptoms over time was slower with greater CPAP use, however this association was not statistically significant. Additionally, TUG scores stabilized in PD patients with treated OSA, whereas they deteriorated in the two other groups. This suggests that OSA can impact not only cognitive function in PD but also motor outcomes. Whether OSA affects the underlying PD neurodegenerative process remains to be determined. 

## 3. Pathogenesis of Cognitive Impairment in OSA

**Figure 1:**
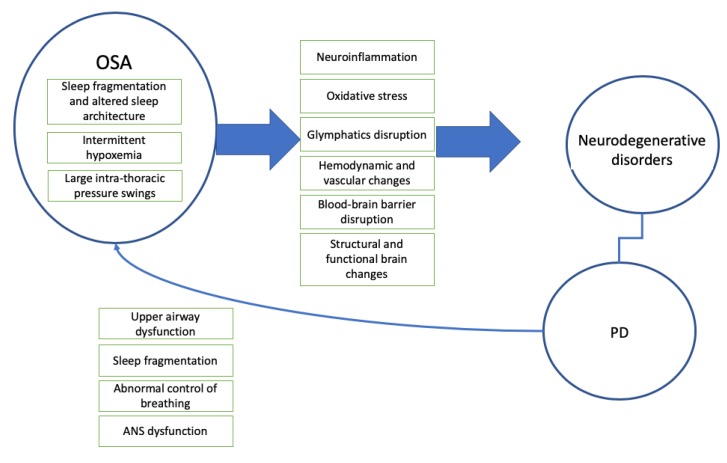
The relationship between obstructive sleep apnea, neurodegenerative disorders, and Parkinson’s disease. OSA, obstructive sleep apnea, and PD, Parkinson’s disease.

The pathophysiologic mechanisms involved in the effects of OSA on the brain remain poorly characterized but are likely complex and multifactorial. Proposed mechanisms, further described below, include intermittent hypoxemia, sleep fragmentation and changes in sleep architecture, hemodynamic and vascular changes, blood–brain barrier disruption, and abnormal waste clearance, changes in synaptic plasticity, which lead to structural and functional brain changes (Figure 1) [51]. 

OSA and dementia share certain risk factors such as age, sex, cardiovascular comorbidities, and genetic background [52,53,54,55,56]. Nevertheless, OSA produces structural and functional brain abnormalities that can be quite similar to neurodegenerative diseases, suggesting the potential for synergistic effects [57]. OSA has been linked to decreased gray matter in the hippocampus, cingulate, and cerebellum, as well as the temporal, frontal, and parietal lobes [14,58,59,60,61,62] and OSA treatment with CPAP was shown to increase gray matter volume in the hippocampal, thalamic, and frontal areas [58,63,64]. Conversely, some studies have found that hypoxemia in OSA is associated with gray matter hypertrophy and thickening [65,66,67,68]. In one study, these changes were also associated with measured respiratory disturbances and sleep fragmentation [65]. In the latter, the authors suggested that gray matter hypertrophy could result from cerebral oedema and sign an early or “presymptomatic” stage of neurodegeneration in OSA that could evolve and progress to atrophy if left untreated. Indeed, oxidative stress and fluctuations in cerebral blood flow induced by intermittent hypoxemia, sleep fragmentation, and respiratory disturbances are known to induce cerebral edema [69,70,71]. Gray matter hypertrophy and thickening were found to respond to CPAP therapy [72]. Furthermore, with the emergence of functional neuroimaging, it has been revealed that OSA decreases brain activation in cingulate, frontal, and parietal regions when performing memory and sustained vigilance tasks, as well as decreased connectivity in similar regions [14,61,73,74,75]. 

Glucose homeostasis and insulin regulation is increasingly being recognized as a risk factor for cognitive impairment [76]. In the general population, higher fasting blood glucose and reduced glucose metabolism were associated with poor cognitive function and cerebral atrophy [77,78]. In a study conducted on middle-aged individuals looking at biological correlates of cognitive impairment, glucose metabolism was strongly and inversely associated with executive functions [79]. Similar work on the proteomic signature of cognitive impairment in postmenopausal women with OSA also identified insulin as one of the biomarkers involved in the development of cognitive impairment [80]. Insulin resistance and diabetes mellitus (type 2 diabetes) is prevalent in patients with OSA, where it is estimated that 15% to 30% are affected [81]. In the non-diabetic, OSA itself is associated with increased insulin resistance and treatment with CPAP in the prediabetic improved glucose metabolism [82,83]. Thus, effects on glucose metabolism could represent a mechanism whereby OSA contributes to cognitive dysfunction [84]. 

OSA is associated with chronic changes in sleep architecture such as sleep fragmentation, decreased slow-wave sleep (SWS), and rapid-eye movement (REM) sleep, as well as possible changes in sleep spindles and K-complexes [85,86,87]. Studies show that these constituents of normal sleep play specific roles in neurocognitive function such as memory consolidation and vigilance by promoting neurogenesis and synaptic plasticity [88,89,90,91,92]. Furthermore, sleep fragmentation appears to be closely related to cognitive outcomes and to predicting episodic memory deficits [25,93]. One study also found that the arousal index and percentage of respiratory arousals negatively correlated with cortical thickness of certain areas in the prefrontal and parietal cortices in male patients with severe OSA [62]. Moreover, reduced neuronal excitability in the locus coeruleus was observed in animal models in response to sleep fragmentation and data suggests that OSA could reduce its noradrenergic neuronal population [94,95]. The locus coeruleus is an area of the brain engaged in synaptic plasticity, motor control, and other functions that have been linked with cognitive decline in the elderly [96]. Studies show that lower neuronal density and decreased connectivity in the locus coeruleus is associated with lower baseline level of cognition, MCI, and faster cognitive decline [97,98]. The locus coeruleus also appears to be implicated in PD pathophysiology, especially with regards to cognitive dysfunction and its involvement seems to precede that of the substantia nigra [96,99]. 

Intermittent hypoxemia has also been implicated in cognitive dysfunction, with animal as well as human studies linking hypoxemia to reduced cognition, especially in domains such as attention, vigilance, and executive function [100,101,102,103]. Possibly, mechanisms include oxidative stress and neuroinflammation in a similar fashion to ischemia and reperfusion injury [104,105]. Intermittent hypoxemia elicits a proinflammatory response through increased expression of mediators such as NADPH oxidase, iNOS, and production of the proinflammatory transcription factor, NF-κΒ [106,107,108]. Markers of systemic inflammation, such as TNF-α, interleukins-6 and -8, and C reactive protein, are elevated in OSA. This raises the possibility that OSA-induced systemic inflammation could contribute to neuroinflammation [109,110]. OSA-related intermittent hypoxemia has also been shown to increase numerous products of oxidation, notably reactive oxygen and nitrogen species [105]. Injury resulting from oxidative stress has been implicated in the pathogenesis of cognitive decline in the elderly, as well as in neurodegenerative diseases [111]. In animal models, exposure to intermittent hypoxia resulted in increased astrocyte and neuronal apoptosis in the frontal cortex and hippocampal regions, reduction in striatal norepinephrine concentration, and injury to specific catecholaminergic neurons in the periaqueductal grey matter and locus coeruleus [95,112]. In humans, OSA with hypoxemia has been associated with delayed peripheral nerve conduction, which is partially reversible with OSA treatment [113,114]. Interestingly, intermittent hypoxemia has also been linked to dysfunction of the blood–brain barrier, an important element in maintaining brain homeostasis. This is possibly due to alteration in microvascular permeability resulting from oxidative stress and systemic inflammation [115]. 

The exact role of intermittent hypoxemia in neurodegenerative diseases remains uncertain. In certain preclinical studies, on the one hand, hypoxemia seems to exert a neuroprotective effect possibly due to an effect of ischemic preconditioning [116,117]. On the other hand, one study conducted on elderly women with OSA found that risk of mild cognitive impairment and cognitive decline was associated with a higher oxygen desaturation index [23], although another study did not reach the same conclusion, possibly because it included mostly mild to moderate OSA [25]. One possible explanation for the discrepant findings concerning the impact of hypoxemia on brain function could relate to different patterns of chronic intermittent versus more sustained hypoxemia, as well as the severity of hypoxemia [104,118,119]. 

Another emerging concept in the pathogenesis of brain dysfunction due to OSA is one of abnormal waste clearance. The glymphatic system is a recently described waste clearance system involved in the transport of cerebrospinal fluid along the brain parenchyma and perivascular spaces to reach the cervical lymphatic system [120]. This system is mainly activated during sleep [121]. Because glymphatics participate in waste clearance through exchanges with the interstitial and cerebrospinal fluid, this system can deal differently with extracellular (B-amyloid and tau protein) and intracellular (alpha-synuclein) proteins [122,123]. Thus, sleep fragmentation due to OSA could alter this homeostatic system and predispose it to protein accumulation and neurodegenerative changes. Furthermore, abnormalities in water and solute fluxes throughout the brain due to intermittent hypoxia and hemodynamic changes related to OSA could also potentially contribute to glymphatic dysfunction. Abnormal glymphatic function has been suggested to contribute to the accumulation of abnormal proteins (β-amyloid, α-synuclein, etc.) implicated in the progression of neurodegenerative disease [124,125]. Moreover, there is some suggestion that alpha-synuclein and other associated proteins could be expressed in a rhythmic, or circadian, fashion [126,127]. Sleep fragmentation could, therefore, possibly potentiate protein aggregation by disruption of these regulating mechanisms, although this has not been studied. 

## 4. OSA Pathogenesis in PD

OSA is estimated to occur in 20% to 60% of PD patients [13]. Its prevalence in PD varies substantially between studies with some having found a higher prevalence [128,129] and others a lower prevalence of OSA in PD patients as compared with healthy controls [41,129,130,131]. The latter is possibly due to limitations such as small sample size, possible selection or participation bias, and more importantly, variable definitions of hypopneas. Previous publications have demonstrated that the definition of sleep-related breathing events, notably hypopneas, can profoundly influence the estimated prevalence of OSA [132,133]. Studies from our laboratory and others indicate that OSA in PD patients manifests principally as hypopneas associated with arousals rather than oxygen desaturation. OSA in PD is associated with a lower desaturation index, higher mean and nadir oxygen saturation, and less time spent with an oxygen saturation below 90% throughout the night as compared with patients with OSA without PD [134,135]. This is likely due to the OSA pathophysiology in this population discussed above, and in part to a lower body mass index (BMI), especially in advanced PD [129,135]. Thus, studies in which the scoring of hypopneas was based only on oxygen desaturation could have missed non-desaturating obstructive events associated with microarousal [10]. Moreover, there can be different patterns of OSA according to the timing of presentation. In some cases, OSA can antedate the development of PD as it is frequent in the general population. It is conceivable that with weight loss, there could be an improvement in such cases. However, as discussed above, OSA can develop in the course of PD, possibly depending on PD subtype or pattern (e.g., tremor-dominant vs. postural instability and gait difficulties) and can worsen as PD progresses [136]. Therefore, estimates of OSA prevalence in PD could vary considerably depending on the patient population studied.

**Figure 2:**
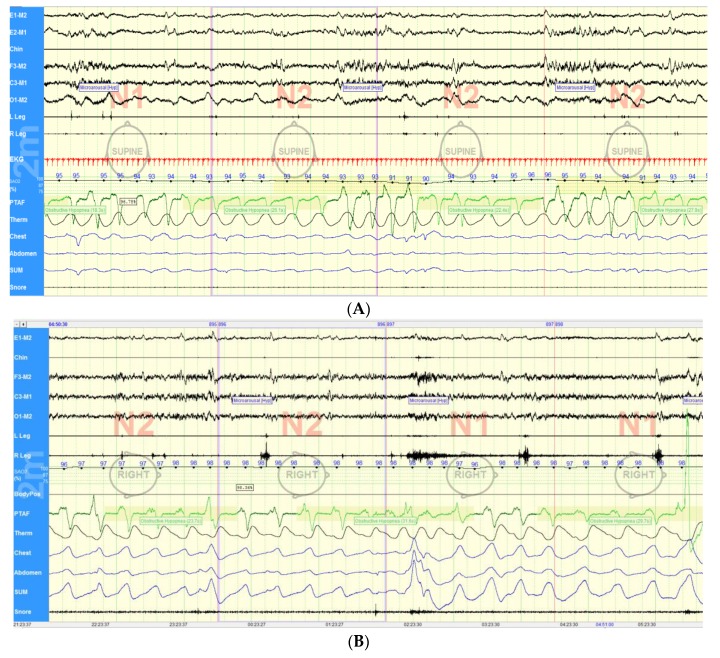
Patterns of upper airway obstruction in Parkinson’s disease. (**A**) Obstructive hypopneas associated with microarousals and oxygen desaturation in a patient with Parkinson’s disease and obstructive sleep apnea. (**B**) Upper airway instability in a patient with Parkinson’s disease resulting in obstructive breathing. PTAF, pressure transducer airflow and Therm, thermistance. Chest and abdomen refer to the respective position of the bands used to detect respiratory efforts and SUM correspond to the sum of the chest and abdominal bands’ signal.

Upper airway obstruction in sleep, the defining element of OSA, results from sleep-related reductions in upper airway dilator muscle tone. In contrast to OSA patients from the general population, upper airway dysfunction has been reported during wakefulness in PD, with two major subtypes described (Figure 2). The first consists of oscillations of glottic and supra-glottic structures, often at a frequency similar to PD tremor, which can manifest as a “saw-tooth” pattern on spirometry [137]. The other subtype exhibits a more obstructive pattern on spirometry due to upper airway muscle instability [138]. Upper airway obstruction during wakefulness can respond to levodopa [139,140,141,142,143]. PD-related upper airway dysfunction can contribute to a sleep-associated decrease in upper airway dilator activity and stability, further predisposing to OSA. Supporting that hypothesis, our group recently reported that night-time treatment with long acting levodopa was associated with reduced OSA severity in PD patients [144]. Additionally, as a disease progresses, motor disability and rigidity become more prominent and significantly impair mobility. Studies have shown that, with time, PD patients have decreased changes in body position at night and more supine sleep than matched controls, which could further predispose to worsening of OSA [145,146].

Impaired ventilatory control, either central or peripheral, has been suggested in PD [147]. The Braak hypothesis for PD progression proposes an early involvement of the brainstem, where respiratory centers and central chemoreceptors lie [148]. Although it is difficult to directly visualize respiratory centers, a study using diffusion tensor imaging has shown initial brainstem and subcortical impairment in de novo PD, which, subsequently, progressed to involve more extensive cortical regions [149]. These imaging studies support findings of an abnormal ventilatory response to hypoxia and hypercapnia in PD patients, despite normal pulmonary function [147,150,151]. Although hypoventilation is rare in PD, abnormal chemoreceptor responses and signalling could contribute to respiratory instability [152,153]. This can be particularly important in non-REM sleep where breathing depends predominantly on chemical drive. Autonomic dysfunction could also be involved in the abnormal feedback to the respiratory centers, and thus predispose to sleep disruption and sleep-disordered breathing [154]. In familial dysautonomia, the ventilatory response to hypercapnia and hypoxemia is substantially impaired [155,156]. This population exhibits a high prevalence of sleep-disordered breathing, with an estimated 85% having some degree of OSA [157]. In PD, abnormal accumulation of alpha-synuclein in the autonomic control pathways and loss of peripheral noradrenergic neurons leads to disruption of the parasympathetic and sympathetic balance and ultimately to a decreased sympathetic tone [158]. Heart rate variability, a common noninvasive method used to evaluate the ANS, is reduced progressively as PD disease severity evolves [159]. However, treatment with dopaminergic agonists also influences sympathetic tone [160,161]. Conversely, dysregulation of the autonomic nervous system (ANS) is also found in OSA, as well as in neurodegenerative diseases [162]. Increase in sympathetic tone associated with baroreflex and chemoreflex changes has been observed in OSA and often persists beyond the sleep period [163,164].

Sleep fragmentation itself can also predispose to respiratory disturbances. Transitions between sleep stages, such as wakefulness to sleep, are associated with a modification in respiration pattern [165]. These changes are usually transient, although in patients with a low arousal threshold, a modest fluctuation in breathing can trigger an arousal. Arousals from sleep following a respiratory event lead to hyperpnea and hypocapnia, which in turn can trigger another respiratory pause upon return to sleep, triggering a cycle of respiratory instability, further promulgating OSA. Sleep fragmentation and dysfunction occur as part of PD and are thought to be multifactorial, due in part to dysfunctional sleep circuits but also to medications and comorbidities. Thus, the intrinsic sleep fragmentation in PD could also be factor in the propensity for OSA in this population.

### Impact of REM Behavior Disorder on PD Manifestations and OSA

REM behavior disorder (RBD) occurs when there is loss of normal REM sleep-induced muscle atonia resulting in complex motor behaviors during REM sleep [166]. Although RBD may be idiopathic, it frequently evolves towards neurodegenerative diseases such as PD, where it has an estimated prevalence of 30% to 50% [167,168,169,170]. A recent systematic review and meta-analysis of longitudinal studies (nearly 3900 patients) estimated that 32% of patients with RBD would convert to neurodegenerative diseases after a mean follow up of 4.75 (±2.73) years and that almost half (44%) converted to PD with long-term follow up [171]. The risk of conversion was evaluated to be as high as 90% at 14 years, consistent with another study in which 38% and 81% of patients developed parkinsonism of dementia at 3.7 (±1.4) and 14 years of follow up, respectively [172,173].

Since upper airway muscle atonia is important in the pathogenesis of OSA, it has been hypothesized that RBD can exert a protective effect and reduce OSA severity. However, evidence is discordant. One study found that patients with the combination of PD, OSA, and RBD had a higher nadir oxygen saturation throughout the whole night and during REM sleep [37]. This is supported by other studies which have also observed a lower apnea-hypopnea index in patients with RBD [174,175]. However, another study reported an increased apnea-hypopnea index in PD patients with OSA and polysomnographic features of RBD [41].

The presence of RBD can influence the expression of motor and non-motor symptoms in PD [169]. Motor manifestations in PD with RBD seem to differ from PD without RBD, exhibiting a non-tremor predominant phenotype and perhaps more postural instability and falls [168,176,177,178,179,180]. In most studies, a combination of RBD and PD is associated with more excessive daytime sleepiness, worse performance on neurocognitive screening tests (MMSE and MOCA), and impairments in specific neurocognitive domains, especially attention and vigilance, executive functions, and memory impairments [37,181,182,183,184]. This is supported by one study showing that 63% of patients with PD and RBD had mild cognitive impairment (MCI) as opposed to 33% with isolated PD and 33% with idiopathic RBD [183]. Furthermore, studies suggest that RBD and OSA together can act synergistically to increase cognitive dysfunction in PD [37,175]. Interestingly, our group found that OSA treatment with CPAP improved RBD symptoms in a cohort of mostly early stage PD patients [185]. Conversely, in a large cohort study OSA was not found to be a predictor of conversion from idiopathic RBD to parkinsonism or dementia [186]. This multicenter study did not have a harmonized protocol for OSA testing, which varied significantly between centers, and information on OSA treatment was not provided.

Other lines of evidence, such as imaging studies, suggest that RBD and OSA could have an additive impact on brain dysfunction in promoting deterioration of cognition. One recent study found that RBD is associated with changes in brain morphology similar to those found with OSA, namely cortical thinning in the frontal, temporal, and parietal cortices and the hippocampal region [187]. Earlier studies observed volume loss in the temporal lobes, cingulate, thalamus, and posterior regions of patients with PD and RBD [188,189,190,191]. However, most of these studies lacked a robust diagnostic method for RBD and comparisons with healthy controls. More recently, one study performed whole brain mapping by combining multiple MRI techniques in PD patients with a PSG-proven diagnosis of RBD and found cortical thinning in the frontal and temporal cortices, as well as subcortical structures. However, it remains unknown whether these changes are secondary to RBD or are a marker of the neurodegenerative process causing RBD. Nevertheless, although no clinical or preclinical studies have specifically evaluated the combination of RBD and OSA on brain atrophy or function, it is theoretically conceivable that the presence of RBD and OSA together could contribute in a synergistic fashion to the development and hastened progression of PD.

## 5. Treatment of OSA in Neurodegenerative Diseases: Benefits and Pitfalls

In the general population with OSA, the effect of CPAP on neurocognitive function has been variable and incomplete, with some neurocognitive domains unaltered by treatment whilst others improve [192,193]. This is likely stems from variability in study designs including patients with different degrees of cognitive deficit, different OSA severity, lack of statistical power, variations in duration of treatment or poor compliance with treatment, and a lack of consistency in neurocognitive testing procedures. Some have suggested that cognitive dysfunction related to excessive sleepiness is readily reversible, whereas the persistent deficits could be due to irreversible brain damage induced, in part, by chronic intermittent hypoxia [194]. However, both sleep fragmentation and intermittent hypoxemia can cause excessive daytime sleepiness and it is methodologically challenging to dissociate the impact of excessive daytime sleepiness on neurocognitive performance from other variables [195,196]. Moreover, in animal studies, intermittent hypoxia has been found to lead to hypersomnolence due to damage to wake-promoting brain regions [197]. In addition, chronic sleep fragmentation could also lead to irreversible changes in brain structure or function [198].

In 2013, a systematic review and meta-analysis of 35 studies (nearly 1000 patients), evaluated patients with varying degrees of OSA before and after CPAP treatment regarding five domains of executive functions (shifting, updating working memory, inhibition, generating new information, and fluid reasoning). Moderate to severe impairment across all five domains was present at baseline and improved with treatment [193]. However, this study did not include the APPLES trial, a large multicenter RCT evaluating CPAP versus sham CPAP on three domains of cognitive function in OSA (attention and psychomotor function, learning and memory, executive and frontal lobe function) [199]. In this study, there was a difference in executive function favoring the CPAP group at two months, but no significant benefits on cognition were apparent at six months, despite significant improvement in subjective and objective sleepiness (especially in the severe OSA subgroup). Part of the explanation could be that patients with a MMSE score ≤ 26 (normal cut-off in healthy adults) were excluded, therefore leaving little room for further improvement. However, a study in older patients with near normal cognition has observed significant improvement in cognitive function with therapy [200]. The relatively young age (mean 52.2 and 50.8 years old in the active and sham PAP therapy groups, respectively) and recruitment through advertisements, and not from a clinical OSA population, could have resulted in a participant group with greater “cognitive reserve” [18] less prone to effects of OSA in the APPLES study.

As the detrimental effects of OSA on cognition could be more apparent in individuals with reduced baseline cognitive function or who are at risk of cognitive impairment, for example due to age or neurocognitive diseases, response to treatment could be greater in those populations. A randomized study in patients over 65 years old showed that treatment with CPAP and conservative care improved episodic and short-term memory, executive functions, mental flexibility, and demonstrated improved connectivity and less cortical thinning on neuroimaging studies as compared with conservative care alone [201]. However, the PREDICT trial, a multicenter RCT conducted in patients 65 years or older with incident OSA, did not share these conclusions [202]. Indeed, despite improvement in sleepiness, patients treated with CPAP did not show improvement in cognitive function as compared with best supportive care. However, the adherence to CPAP could have been insufficient (mean use < 2 h/night). A smaller scale study emphasized the importance of longer CPAP usage on cognitive function in patients over 55 years of age with normal cognition (MMSE > 25) [200]. They observed greater improvement of memory, learning, and psychomotor speed in compliant CPAP users (mean 8.5 h per night) as compared with noncompliant users (mean 3.9 h per night). Finally, treatment duration can be a key factor. Indeed, most studies which found improvement in cognition with CPAP treatment were conducted for more than one to three months [192,203]. Interestingly, one study conducted in treatment naïve patients with OSA observed not only an improvement in neurocognitive testing after three months of treatment but also an improvement in white matter integrity on diffusion tensor imaging at three months [203] which was more appreciable at 12 months. Overall, improvements on neurocognitive testing were correlated with improvements in white matter integrity. Another long-term study (mean 18.2 ± 44 months) observed that longer treatment with CPAP yielded broader increments in regional cortical volume [64]. Consistent with these results, a more recent adherence study also found that one year of CPAP improved cognition and could slow cognitive decline in patients with OSA and mild cognitive impairment [204]. These findings further support the benefits of adequate treatment duration and also emphasize the importance of ageing in the susceptibility to OSA effects on cognitive dysfunction and response to treatment.

**Figure 3:**
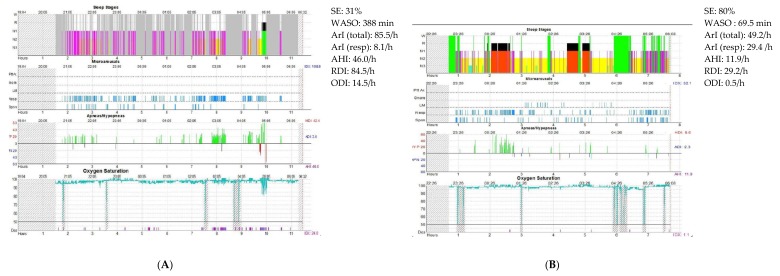
Changes in sleep architecture following treatment with automatic positive airway pressure in a patient with Parkinson’s disease and severe obstructive sleep apnea. The graphs represent (from top to bottom): sleep stages, microarousals, respiratory events, and oxygen saturation. (**A**) Polysomnography before treatment that shows reduction in sleep efficiency (SE), substantial sleep fragmentation with many arousals, increased wake after sleep onset (WASO), reduced slow wave sleep, and prolonged REM latency with only one REM period occurring at the end of the night. (**B**) Polysomnography performed on automatic positive airway pressure. It shows improvement of SE, WASO, arousals, and overall sleep architecture with increased proportion of slow wave and REM sleep and normal REM latency (60 min). AHI, apnea-hypopnea index; LM, leg movement; RDI, respiratory disturbance index; and ODI, oxygen desaturation index as defined by a drop in pulse oxygen saturation of 3 % or greater.

Positive pressure therapy has also shown benefits in patients with neurodegenerative diseases (Figure 3). One study evaluated 39 community-dwelling patients with AD and OSA treated with six weeks of CPAP versus three weeks of sham CPAP followed by three weeks of CPAP. They found a significant reduction in sleepiness after three and six weeks of active therapy as opposed to no change with sham CPAP [205]. Another three-week sham-controlled CPAP trial conducted in patients with mild-moderate AD found no improvement in MMSE and no change in neurocognitive functioning, assessed by a composite neuropsychological score, between groups after three weeks of treatment versus sham, possibly due to insufficient statistical power [206]. However, the composite neuropsychological score improved from baseline in combined therapeutic periods from both groups (sham group subsequently also treated with active CPAP for three weeks). Patients with sustained CPAP use in an open-label follow up (mean 13.3 months, range = six to 21 months) showed less deterioration of their global cognition, especially regarding executive functions, as compared with the untreated group, and less deterioration or even improvement in depressive symptoms, daytime sleepiness, and subjective sleep quality [207]. Treatment with CPAP also improved caregivers’ satisfaction as they reported improvement in their sleep quality and mood. Notwithstanding the encouraging results, CPAP therapy is a challenge in AD patients. In the trial above, recruitment was arduous and 25% (seven/27 patients, six at three weeks and one at six weeks) and 20% (five/25 patients, two at three weeks and three at six weeks) of the therapeutic and placebo CPAP groups, respectively, discontinued treatment [206].

In PD, a short-term randomized crossover trial reported improvement of objective daytime sleepiness, sleep quality, AHI, and oxygen saturation with CPAP treatment of OSA [208]. PD patients were able to use their device despite motor dysfunction and exhibited good adherence to treatment (88% of the night with a mean nightly use of 5.2 h) [208]. OSA was associated with significantly lower MoCA and MMSE at baseline, but no significant improvement was found after three or six weeks of CPAP. Absence of benefits could be related to smaller sample size, lower severity of OSA, and the short duration of study. However, a more recent cohort study by our group evaluating CPAP treatment over 12 months found improvement in non-motor symptoms overall, subjective sleep quality, anxiety, and cognitive function [209]. Cognitive improvement was found without a significant change in subjective daytime sleepiness, suggesting independent pathways can mediate these effects of OSA. Furthermore, improvements were noted despite modest nightly CPAP use (3.5 mean hours per night). Hence, it is possible that a longer duration of treatment is necessary for modification of specific symptoms, particularly cognitive function. Moreover, to date, there have been no long-term randomized controlled trials assessing whether OSA treatment impacts the course or rate of progression of PD.

Treatment with CPAP in the PD population poses challenges beyond those in the general population and the elderly. Cognitive deficits and motor disability can render device handling difficult. Nocturia, anxiety, and RBD are additional potential challenges. One study found that only 51% of patients diagnosed with OSA agreed to a CPAP titration, and in those who tried CPAP, the attrition rate was 75% with most patients abandoning within the first three to five weeks of treatment initiation due to intolerance [210]. This was a fairly elderly group with a low education level on average, who received little support at treatment initiation, factors that could have accounted for the high rate of CPAP abandonment. In our cohort, 39 of 46 (85%) patients with PD and OSA agreed to try CPAP, and at 12 months, 21 (54%) were still regular users [209]. Numerous factors predicting adherence, or non-adherence, to CPAP have been identified [211]. In addition to addressing side effects, factors that could help improve adherence include active education of the patients, inclusion of the caregiver in the education and adherence process, early and repetitive follow-up of CPAP usage by a competent healthcare provider after treatment initiation, available resources for troubleshooting, and clinical follow up with the sleep team [211,212,213]. Other barriers to CPAP use, including psychological or social factors, should be sought and addressed as some patients could benefit from more intensive behavioural therapy interventions or additional support from providers [214,215]. Studies evaluating specific interventions to help with CPAP use in neurodegenerative disease and development of therapies that are effective, as well as easy to use, are needed in order to better tailor therapy to the specific challenges this population faces.

Alternatives to PAP therapy have been less well studied in PD. However, in one recent pilot study 10 PD patients who refused or were intolerant of PAP therapy were treated with mandibular advancement devices (MAD) [216]. MAD therapy was associated with improved sleep complaints, and reduced apnea-hypopnea and oxygen desaturation indices. Patients also demonstrated better compliance with MAD than PAP therapy and did not experience more adverse events as compared with matched patients on CPAP. Some side effects of MAD, such as hypersalivation or xerostomia and discomfort, could be of relevance and limit its use in PD patients. Larger randomized studies are needed to confirm these results and assess feasibility and safety of MAD in this population.

Upper airway obstruction can also respond to levodopa [139,140,141,142,143]. Supporting this hypothesis, our group found that night-time treatment with long acting levodopa was associated with reduced OSA severity in PD patients, suggesting it could be a potential alternative to PAP therapy [144]. Prospective studies to better understand the effect and the role of long-acting levodopa in OSA treatment are needed. Conversely, dopaminergic agonists can contribute to upper airway dysfunction by inducing dyskinesias [217]. Prospective studies are needed to better understand the efficacy of long-acting levodopa in OSA treatment.

## 6. Conclusions

OSA is associated with functional and structural changes in the brain which can be particularly detrimental to patients with neurodegenerative diseases, leading to exacerbation of non-motor and motor dysfunction in this population. Further studies are needed to better characterize the impact of treatment on patient-centered outcomes, especially in the context of a difficult-to-treat population. Strategies to improve adherence to CPAP are needed, focusing on the specific challenges in this patient group, and alternate treatment options should be explored. Finally, disease modification trials are required to determine whether OSA treatment, in addition to improving symptoms, can impact the course of neurodegeneration over time.
